# Potent Bioactivity of Endophytic Fungi Isolated from *Moringa oleifera* Leaves

**DOI:** 10.1155/2022/2461021

**Published:** 2022-12-15

**Authors:** Bushra Rehman, Sumera Afzal Khan, Muhammad Hamayun, Amjad Iqbal, In-Jung Lee

**Affiliations:** ^1^Centre of Biotechnology and Microbiology, University of Peshawar, Pakistan; ^2^Department of Botany, Garden Campus, Abdul Wali Khan University Mardan, Pakistan; ^3^Department of Food Science & Technology, Garden Campus, Abdul Wali Khan University Mardan, Pakistan; ^4^Department of Applied Biosciences, Kyungpook National University, Daegu 41566, Republic of Korea

## Abstract

Plant species are known to harbor large number of endophytes, which stays in plant tissues as symbionts. These endophytes secrete large array of bioactive compounds that have potency against certain diseases with no side effects. We have collected leaf samples of the *Moringa oleifera* plant from the Pakistan Forest Institute, Khyber Pakhtunkhwa, Pakistan for the isolation of beneficial endophytes. The strains isolated from the leaves of *M. oleifera* were coded with MOL and tested for antimicrobial, antifungal, germicidal, phytotoxic, insecticidal, cytotoxic, and anti-inflammatory activities. The isolates, MOL1, MOL16, MOL19, and MOL21, possessed antibacterial activity against *Staphylococcus aureus*, whereas MOL7 inhibited 55% of the growth of *Escherichia coli*. MOL3 inhibited the growth of *E. coli*, S. *aureus*, and *Pseudomonas aeruginosa*. The strains, MOL1 and MOL7, showed antifungal activity against *Candida albicans* and *Saccharomyces cerevisiae*, while the strains, MOL11 and MOL17, showed activity against *Verticillium chlamydosporium*. The isolates, MOL3, MOL7, MOL9, MOL15, MOL17, MOL18, and MOL19, inhibited the growth of *Lemna minor* (duckweed) at 100 *μ*g/ml. MOL2 exhibited strong activity in the brine shrimp assay, while MOL1, MOL2, MOL5, MOL6, MOL12, MOL17, MOL19, and MOL20 showed insecticidal, and MOL3 demonstrated larvicidal and antileishmanial activity. The isolated potent endophytes were identified as *Aspergillus, Penicillium, Fusarium, Tricoderma, Rhizoctonia, Mucor, Alternaria, Pestalotiopsis, Acremonium*, *and Cladosporium* through morphological and microscopic characteristics of the colonies.

## 1. Introduction

Fungal endophytes live inside tissues of their host plants and had been reported for beneficial effects on their host. Endophytes became known to human knowledge since last century. However, there is also a hypothesis that the evolution of endophytes dates back to evolution of land plants [1]. Recently, endophytes are gaining interest as sources of biologically active secondary metabolites [2]. Exploration of the endophytic microflora is a step towards saving medicinally important plants from extinction. It encourages to harvest medicinally important metabolites from endophytic microbiota instead of the host plant species [3].

*Moringa oleifera* commonly known as Drumstick, Ben tree, Magic tree, or Suhanjna (Urdu) is a tree having huge medicinal importance. Pharmacological studies showed that the ethanolic extract of *M. oleifera* leaves have antihypertensive (hypotensive) activity [4]. Similarly, alcoholic extracts of seed kernels of *M. oleifera* exhibited antiasthmatic activity [5], whereas the aqueous extract of leaves demonstrated antidiabetic activity [6]. In another study, the hepatoprotective activity was noticed in leaves and seeds [7] and anticancerous activity was noted in ethanolic extract of *M. oleifera* seeds [8, 9].

Fungal endophytes are known to produce a large number of bioactive compounds that are beneficial for humans as well as plants [10–15]. Literature showed that the minimum inhibitory concentration (MIC) values of the chloroform extract of endophytic *Aspergillus fumigatus* ranged from 0.05 to 0.5 mg/ml, and the viable cell count study revealed its bactericidal activity. Mutagenicity and MTT assay disclosed that the crude extract of the said endophyte is neither cytotoxic nor mutagenic showing that it is biosafe [16]. Carbungco et al. [17] isolated a total of 24 endophytic fungi belonging to the genera *Fusarium, Xylaria, Pestalotiopsis, Aspergillus, Nigrospora, Stachybotrys, Rhizoctonia,* and *Macrophomina* from *M. oleifera* leaves. Fungal endophytes of *Moringa* and their metabolites had not been explored in any locality of Pakistan. The compounds produced by endophytic fungus of *M. oleifera* could be an alternative source for human welfare. The current study not only aimed at isolating and identifying the endophytic fungi from the leaves of *M. oleifera* but also searching for the potent yet novel bioactive compounds from this medicinal plant.

## 2. Materials and Methods

### 2.1. Collection of Samples

Mature trees of *M. oleifera* (3 to 8 years old) grown in Pakistan Forest Institute, Khyber Pakhtunkhwa, Pakistan were randomly selected for the collection of leaf samples. The collected leaf samples were transferred to the zipper bags, labelled and transported to the microbiology laboratory at Center of Biotechnology and Microbiology, University of Peshawer for further processing. Physical examination was performed for visible symptoms of disease; healthy samples were processed for fungal isolation within few hours after collection.

### 2.2. Isolation of Pure Strains of Endophytic Fungi

The endophytes were isolated using a modified method described by Arnold [18]. Briefly, leaf samples were carefully washed with sterile water and then surface sterilized with 75% ethanol for 30 seconds, followed by soaking in 2% of sodium hypochlorite (NaOCl) solution for 2 minutes. The leaves were finally rinsed thoroughly with sterile distilled water to remove any traces of the ethanol and NaOCl.

Air dried samples were cut in 1 cm pieces aseptically with the help of sterile scalpel, which were then carefully placed on Sabouraud Dextrose agar (SDA) plates, and incubated at 30°C for 7 days. Chloramphenicol was added in the media before pouring in plates to avoid bacterial growth. The plates were thoroughly checked on a daily basis to monitor the growth of endophytic fungi. After the appearance of the fungal hyphae, they were carefully picked and inoculated on fresh labelled plates and slants of Potato Dextrose Agar. Repeated culturing and subculturing resulted in pure cultures. Fungal cultures were then stored at 4°C till further processing.

### 2.3. Cultivation of Endophytic Fungi and Metabolite Extraction

The fungus was cultured by placing agar blocks of actively growing pure culture (3 mm in diameter) in a 500 ml Erlenmeyer flask containing 100 g of rice medium. Incubation was done at room temperature for 3 weeks. Afterward, the fermentation was brought to a halt by the introduction of 500 ml of ethyl acetate into the flask. The Whatman No. 1 filter paper was used to separate the fermented mixture. The metabolite from the fungus was extracted by using ethyl acetate as an organic solvent. Ethyl acetate was then evaporated with the help of a rotary evaporator at 50°C, and the resultant compounds were dried in CaCl_2_ desiccators to yield the crude metabolites. The crude extract was then kept in an Eppendorf tube at 25°C for bioassays.

### 2.4. Identification of Isolated Endophytic Fungi

#### 2.4.1. Morphological Identification

The method of Diba et al. [19] was adopted for the morphological identification of various fungal strains isolated from the leaves of *M. oleifera*. Mycological identification keys and taxonomic description were used to identify the isolated fungi at the genus level [20].

#### 2.4.2. Microscopic Identification

The morphology of spores as well as mycelia of fungal isolates were studied, recognized, and identified by using the lactophenol blue staining and light microscopy [21].

### 2.5. In Vitro Biological Activities

#### 2.5.1. Antibacterial Assay

Pathogenic bacterial strains (*P. aeruginosa, S. aureus, K. pneumonia, E. coli, Enterobacter* sp.) were obtained from the pathology lab of Khyber Teaching Hospital (KTH), Peshawar, Pakistan.

By using the well diffusion method, the crude ethyl acetate extracts of endophytic fungi suspended in Dimethyl Sulfoxide (DMSO) were screened against bacterial pathogens, and the zones of inhibition were measured [22, 23]. Amoxicillin was used as positive control, while dimethyl sulfoxide (DMSO) was used as negative control. Percent inhibition was calculated using following formula:

(1) $$\begin{array}{c} \%\text{Inhibition}=\frac{\text{Linear growth in test }\left(\text{mm}\right)}{\text{Linear growth in standard }\left(\text{mm}\right)}\times 100. \end{array}$$

#### 2.5.2. Antifungal Activity

*Verticillium chlamydosporium, Candida albicans, Saccharomyces cerevisiae, Pneumocystis pneumonia* were obtained from the Plant Pathology Department, Agriculture University, KP, Pakistan.

The antifungal activity was performed as per reported procedure of Ahmad et al. [24]. Exactly, 24 mg/ml stock solution of test sample was prepared by using DMSO. Slants preparation was done by using about 5 ml of SDA medium. Then, from stock solution, 66.6 *μ*l of the sample was transferred to test tubes containing 7 days old fungal culture, and the tubes were incubated for 7 days at 25 ± 1°*C*. After seven days of growth, results were compared with controls, and percent inhibition was calculated. For positive controls, we used Amphotericin-B for *C. albicans* and Miconazole for other pathogenic fungi [25]. DMSO was used as a negative control.

### 2.6. Phytotoxic Assay

Phytotoxic properties of the isolated endophytes against *Lemna minor* were assessed by adopting the established protocol of Rashid et al. [26]. Briefly, E-medium was prepared and autoclaved at 121°C for 15 minutes. Dimethyl sulfoxide (DMSO) was used to make 20 mg/ml of crude extracts as stock solutions. To each sterilized flask, 20 ml of E-medium was added, followed by addition of eighteen *L. minor* leaves. Paraquat was used as positive control and E-medium without extracts as negative control. The flasks were plugged with sterile cotton and then placed in a plant growth chamber for seven days at 30°C, 9000 Lux and 60% moisture. The fronds were visually examined after seven days of incubation. The percent growth inhibition was calculated as

(2) $$\begin{array}{c} \%\text{Growth inhibition}=\frac{\left(100-\text{number of fronds in test}\right)}{\text{number of fronds in control}}\times 100. \end{array}$$

### 2.7. Cytotoxic Assay (Brine Shrimp Lethality Assay)

The crude extract of fungal metabolites was used to measure the cell lethality against the eggs of brine shrimps (*Artemia salina*). Marine water was prepared by dissolving 38 g of sea salt in 1000 ml of purified H_2_O. The mixture was filtered, and the pH was set to 7.4 in order to facilitate hatching of the shrimp eggs [25].

In a small plastic hatching chamber, the seawater was kept, having dark and light partitioning. Eggs (1 mg) were placed at the dark/shaded side of the chamber, while the hatched shrimp were attracted by the light and moved to the other side. Larvae per ten shrimps were collected and shifted to flasks containing 5 ml of sea water. In 1% DMSO, the crude extracts were made at 20 mg/mL concentration. Plain marine water was used as a negative control, while Etoposide, a cytotoxic drug, was used as a positive control. Magnifying glass was utilized to count the dead shrimps after 24 hours of incubation period [27], and the cytotoxicity was calculated using the following formula:

(3) $$\begin{array}{c} \%\text{Death}=\frac{\text{No}.\text{of dead nauplii }\left(\text{nu}\right)}{\text{No}.\text{of dead nu}+\text{No}.\text{of live nu}}\times 100. \end{array}$$

### 2.8. Insecticidal Assay

The direct contact method, also known as the impregnated filter paper method, was used to determine the insecticidal activity of the fungal crude extracts against the *Callosobruchus analis*, *Tribolium Castaneum*, and *Rhyzopertha dominica*. The insect species were obtained from the National Institute of Food and Agriculture, NIFA, Peshawar. Briefly, crude endophytic fungal extracts in methanol were added to the filter paper in petri plates with the help of sterile micropipette. After overnight evaporation, the insects of the same size and age were transferred to the plates. The plates containing insects were incubated overnight in a growth chamber at 27°C; the humidity was maintained by placing a beaker full of water in the growth chamber [28]. Permethrin was used as a positive control, while methanol was used as a negative control.

The percent mortality was calculated as follows:

(4) $$\begin{array}{c} \%\text{Mortality}=\frac{\text{No}.\text{of insect alive in treatment}}{\text{No}.\text{of insect alive in control}}\times 100. \end{array}$$

### 2.9. Larvicidal Activity

The larvicidal effect of endophytic fungal extracts was tested against *Aedes aegypti* larvae according to the standard protocol of the World Health Organization (WHO) [29]. Crude ethyl acetate extracts of fungi were added to DMSO in order to prepare 200 ppm of the test solution. Larvae (25) of *Ae. aegypti* were shifted to a 250 ml bowl containing 200 ml of the test solution. After 24 hours, larvae with no movement were gently touched to ensure that they were dead. DMSO was used as a negative control, whereas Permethrin was used as a positive control. Percent mortality was calculated with Abbott's formula.

(5) $$\begin{array}{c} \%\text{Mortality}=\frac{\left(\%\text{mortality in test}-\%\text{mortality in control}\right)}{100-\%\text{mortality in control}}. \end{array}$$

### 2.10. Antileishmanial Assay

To 200 *μ*l of test sample (180 *μ*l medium, 20 *μ*l crude extract), a 100 *μ*l of leishmanial culture was added. The mixture was incubated in the Neubauer chamber for 3 days at 22°C. After 3 days of incubation, the number of alive parasites was counted microscopically, and IC50 value was determined using Ezfit 5.03 software [30]. Negative control contained leishmanial cell in plain media, while positive control included media, leishmanial culture, Pantamidine (ICN), and amphotericin B (Fluka).

## 3. Results and Discussion

### 3.1. Isolation of Endophytic Fungi

Incubation of sterile cut leaves of *M. oleifera* on PDA plates resulted in different colored and textured colonies. Pure isolates were obtained by repeated culturing and subculturing of endophytic fungi based on difference in colonies' margins, pigments, growth rate, and morphology. A total of 22 endophytic fungal strains was isolated from the leaves of *M. oleifera*.

### 3.2. Characterization of the Isolated Fungal Endophytes

Isolated endophytic fungi from *M. oleifera* leaves were identified via morphological (microscopic and macroscopic) characteristics (Table S1 and S2 and Figure S1). The identified species were coded as MOL1, MOL2, MOL3, MOL5, MOL7, MOL8, MOL9, MOL10, MOL13, MOL14, MOL19, MOL21, and MOL22. These endophytic fungal isolates were identified on the basis of colony surface, reverse side color, growth rate, margin of the colonies, and microscopic features, such as hyphae, conidiophore, conidia, and fruiting bodies. The isolated species belonged to *Aspergillus*, *Penicillium, Fusarium, Tricoderma, Rhizoctonia, Mucor, Alternaria, Pestalotiopsis, Acremonium* and *Cladosporium* genera (Table S1). Carbungco et al. [17] isolated 24 fungal species from the leaves of *M. oleifera* that belonged to genera *Fusarium, Xylaria, Pestalotiopsis, Aspergillus, Nigrospora, Stachybotrys, Rhizoctonia,* and *Macrophomina*. Similarly, a number of endophytes belongs to *Alternaria, Cladosporium, Colletotrichum, Corynespora, Curvularia, Fusarium, Mucor, Ochrocladosporium, Phomopsis* and *Trametes* have been isolated from the *M. oleifera* gathered from Lombok Island, West Nusa Tenggara [31]. In fact, most of the endophytes that belong to the above-mentioned genera are already known for the production of bioactive compounds. For example, *Penicillium roqueforti* harbors andrastins A-D [32], *Fusarium oxysporum* produces volatile organic compounds [33], and *Aspergillus carneus* releases depsipeptides, marcfortine A, and aspergillicins A–E [34].

### 3.3. Antibacterial Activity

**Figure 1:**
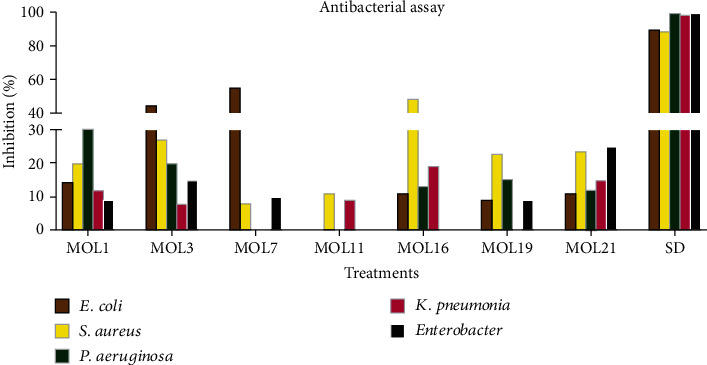
Antibacterial activity of endophytes isolated from *M. oleifera* leaves.

Fungal endophytes are usually acknowledged for the production of antibacterial metabolites, such as 4-hydroxybenzoic acid, gibepyrone D, and indole-3-acetic acid (IAA) against the pathogenic bacteria [35]. In the present study, MOL7 (55%) and MOL3 (45%) exhibited good antibacterial activity against *E. coli*, whereas MOL16 (48%) and MOL1 (30%) showed appreciable activity against *S. aureus* and *P. aeruginosa*, respectively (Figure 1). The isolate MOL1 inhibited the growth of *Enterobacter* (15%)*, E. coli* (20%)*, S. aureus* (12%), and *K. pneumonia* (9%), but their activity was low. Likewise, MOL3 and MOL21 showed activity against all the tested pathogens, but its activity was low against *S. aureus* (27% and 23%)*, P. aeruginosa* (20% and 12%)*, K. pneumonia* (8.8% and 15%), and *Enterobacter* (15% and 25%). The low activity of MOL7 was noted against *S. aureus* and *Enterobacter*, and zero activity was noticed against other tested bacterial pathogens. MOL11 showed low activity against *S. aureus* (11%) and *K. pneumonia* (9%), and MOL16 showed low percent inhibition against *E. coli* (11%), *P. aeruginosa* (13%), and *K. pneumonia* (19%), respectively. MOL19 displayed modest percent inhibition against *E. coli* (9%), *S. aureus* (22%), and *P. aeruginosa* (15%) and inactive against *K. pneumonia* and *Enterobacter* (Figure 1). The extract of the all other isolated endophytes from *M. oleifera* leaves was inactive against the tested pathogenic strains of bacteria (Results not shown). Muhammad et al. [10] observed antibacterial properties of endophytic *Penicillium roqueforti and Trichoderma reesei* isolated from *Solanum surattense*. Best antimicrobial activity of ethyl acetate extract of *P. roqueforti* was observed against *Xanthomonas oryzae*, *Agrobacterium tumefaciens*, *P. syringae*, *and Ralstonia solanacearum*. Similarly, the endophytic strain *T. reesei* displayed highest activity against *X. oryzae*, followed by *A. tumefaciens, P. syringae*, and *R. solanacearum*. In the past, metabolites of endophytic fungi isolated from the leaves of *Indigofera suffruticosa* presented antibacterial activity [36]. In fact, the isolation and purification of potent metabolites from the endophytic fungi have great prospects in plants and human health.

### 3.4. Antifungal Activity

**Figure 2:**
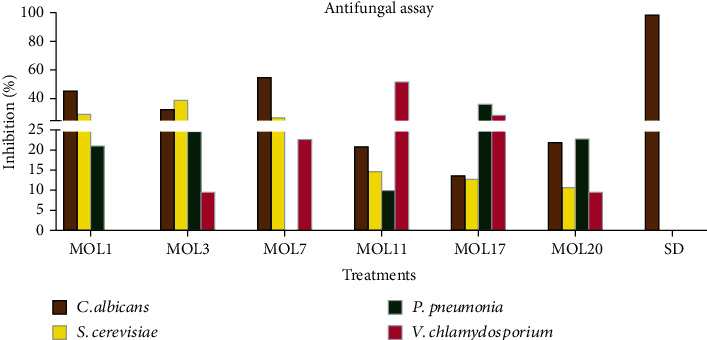
Antifungal activity of endophytes isolated from *M. oleifera* leaves.

MOL3, MOL11, MOL17, and MOL20 demonstrated antifungal activity against all the tested fungal strains (Figure 2). MOL1 showed moderate antifungal activity against *Candida albicans* (46%), *Saccharomyces cerevisiae* (30%), and *Pneumocystic pneumonia* (21%), whereas no activity against *Verticillium chlamydosporium*. The antimicrobial activity of MOL3 against pathogenic *C. albicans* was 33%, *S. cerevisiae* was 40%, *P. pneumonia* was 25%, and *V. chlamydosporium* was 10%. MOL7 showed higher antifungal activity against *C. albicans* (56%), low activity against *S. cerevisiae* (27%) and *V. chlamydosporium* (23%), but no activity against *Pneumocystic pneumonia*. MOL11 showed strong antifungal activity against *V. chlamydosporium*, while moderate to low activity against *C. albicans*, *S. cerevisiae*, and *P. pneumonia*. The effect of MOL17 extracts on percent inhibition against *C. albicans* was 14%, *S. cerevisiae* was 13%, *P. pneumonia* was 36%, and *V. chlamydosporium* was 30%. Antifungal activity of MOL20 against all 4 tested were noticed, but the activity of crude extract was weak (Figure 2). Moreover, the extract of the all other isolated endophytes from *M. oleifera* leaves was inactive against the tested pathogenic strains of fungi (Results not shown). As it is known that endophytes release a broad range of potent compounds, more and more researchers from various fields including health are engaged to evaluate the antimicrobial activity of these fungi [10]. In this perspective, Yu et al. [37] observed antifungal activity of endophytic fungi ty-64 (Oidium sp.) isolated from the *Camellia oleifera* against the pathogenic *C. oleifera* anthracnose. *Aspergillus terreus* is one of the well-studied endophytes that releases hundreds of compounds to support host plant under stress [15]. Quite recently, it was observed that the compounds in the *A. terreus* exudates can inhibit the growth of mucormycosis causing *Syncephalastrum racemosum*, *Mucor racemosus*, and *Rhizopus oryzae* [38]. The potent antifungal activity of endophytic fungi is due to the presence of cytochalasin alkaloids, terpenoids, polyketides, propanoic acid, piliformic acid, cordycepsidone A, ferulic acid, etc. in their exudates [39].

### 3.5. Phytotoxic Activity

**Figure 3:**
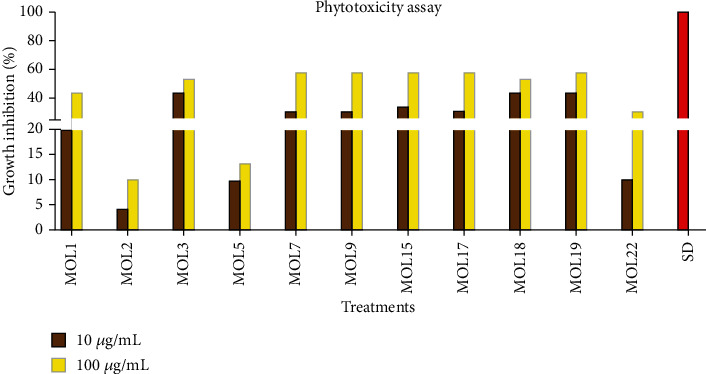
Phytotoxic activity of fungal metabolites from *M. oleifera* leaves.

Focus on endophytic fungal secondary metabolites is growing day by day to discover novel phytotoxic compounds in order to develop bioherbicides. The use of organic weedicides is one of the best substitutes to monitor a vast array of weeds in crops and forests [40–42]. In fact, agricultural productivity in terms of crop yield got reduced around the globe, especially in Pakistan because of weeds. Losses in crops' growth and development caused by weeds have ignored over the years; however, weeds harm the agricultural crops more as compared to the insects and pests [43]. In this study, some of the isolated fungi from leaves of *M. oleifera* exhibited good phytotoxic activity against *Lemna minor* (Figure 3). As expected, the metabolites of the potent strains showed high activity at 100 *μ*g/ml concentration as compared to the 10 *μ*g/ml against *L. minor*. The exudates of isolate MOL3 showed good percent inhibition, i.e., 40% at 10 *μ*g/ml and 50% at 100 *μ*g/ml. MOL7, MOL9, MOL15, MOL17, and MOL19 were among the best strains which inhibited higher than 50% of *L. minor* growth at 100 *μ*g/ml. MOL1 showed 40% inhibition at 100 *μ*g/ml and 20% at 10 *μ*g/ml. The crude extract of endophytic fungal MOL2 and MOL5 isolates demonstrated very weak phytotoxic activity (Figure 3). The exudates of remaining isolates were found inactive against *L. minor* (Results not shown). A large number of endophytes have been reported to release nonvolatile as well as volatile compounds that have proven phytotoxic activity against various weeds [44]. In 2016, Ulloa-Benítez et al. [45] noted the phytotoxic activity of *Hypoxylon anthochroum* extracts against seed germination, seedlings respiration and elongation of roots of *Panicum miliaceum*, *Medicago sativa*, *Amaranthus hypochondriacus*, and *Trifolium pratense*. Further experiments conducted by them unleash the presence of eucalyptol and phenylethyl alcohol. These observations demonstrate that endophytic fungi can be used as a reservoir of bioherbicides or weedicides.

### 3.6. Brine Shrimp Biolethality Assay

**Figure 4:**
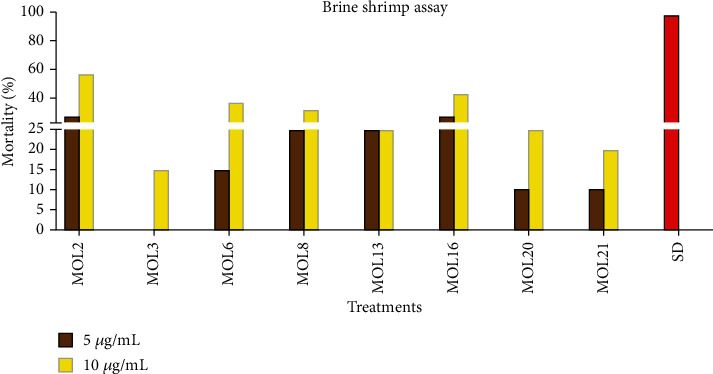
Brine shrimp biolethality assay of leaves endophyts of *M. oleifera*.

Brine shrimp biolethality (BSL) assay was carried out by using crude extracts of endophytic fungi isolated from the leaves of *M. oleifera* (Figure 4). Similar to phytotoxic assay, the BSL assay demonstrated high activity of potent endophytes at high concentration (10 *μ*g/ml) as compared to the low concentration (5 *μ*g/ml). Besides this, great diversity (ranged from 10% to 60%) was observed in the extracts of endophytes that showed activity against the shrimps. The fungal extracts, MOL2, MOL6, MOL8, and MOL16, exhibited high toxicity at 10 *μ*g/ml, while at 5 *μ*g/ml same samples showed mild activity. The extract of fungal endophytes MOL3, MOL20, and MOL21 displayed low percent mortality at 10 *μ*g/ml concentration. Quite interestingly, the sample of MOL13 displayed same activity (25%) at 5 and 10 *μ*g/ml (Figure 4). The extract of remaining fungal endophytes isolated from *M. oleifera* leaves showed no activity in the BSL assay (Results not shown). BSL assay is indeed a basic assay to notice the cytotoxic potential of natural or synthetic drugs; however, for the confirmation of cytotoxicity rigorous experiments, using cell lines is needed. It has been known for years that the synthetic drugs have the capability to fight the cancerous cells, but they possess some unavoiding side effects. To keep health deterring side effects of the synthetic drugs away, an alternative natural remedy with no side effects is required. Endophytes can serve the said purpose very well as it can release Taxol, podophyllotoxin, camptothecin, vinca alkaloids, phomoxanthone a, brefeldin a, altersolanol, macrosporin, etc. [46]. Our results are in close agreement with those of Majoumouo et al. [47], who observed cytotoxic activity in the extracts of *Fusarium oxyporum*.

### 3.7. Insecticidal Activity

**Figure 5:**
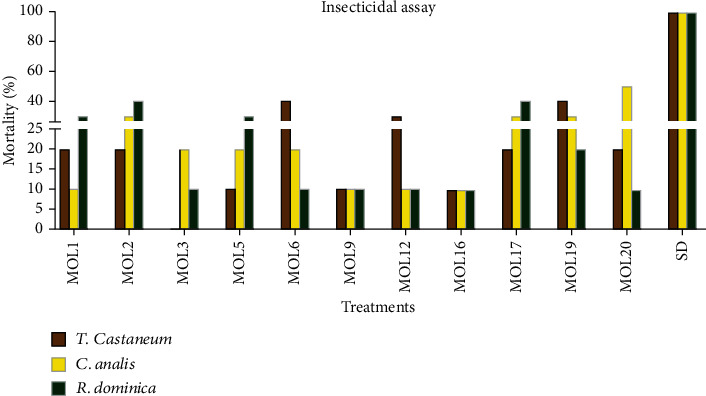
Insecticidal activity of fungal metabolites isolated from *M. oleifera* leaves.

Extracts from endophytic fungi contain numerous compounds, which play a key role in defending host plant species against insects and pests. In this study, the insecticidal activities of fungal extracts isolated from *M. oleifera* leaves were examined against *Tribolium castaneum*, *Callosbruches analis*, and *Rhyzopertha dominica* (Figure 5). High mortality of *T. Castaneum* was recorded for the extracts of endophytic isolates MOL6 (40%) and MOL19 (40%), whereas MOL12 (30%) showed moderate activity. The extracts of isolates MOL1 (20%), MOL2 (20%), MOL17 (20%), and MOL20 (20%) demonstrated low, and MOL5 (10%), MOL9 (10%), and MOL16 (10%) exhibited very low activity against *T. Castaneum*. In case of *C. analis*, 50% of the mortality was caused by the extract of MOL20, followed by MOL2 (30%), MOL17 (30%), and MOL19 (30%). The extracts of MOL3 (20%), MOL5 (20%), and MOL6 (20%) showed weak, and MOL1 (10%), MOL9 (10%), MOL12 (10%), and MOL16 (10%) exhibited very weak anti-*C. analis* activity. Strong mortality was observed in *R. dominica*, when they were given the crude extracts of MOL2 (40%), MOL17 (40%), MOL1 (30%), and MOL5 (30%). On the other hand, MOL19 (20%) presented low anti-*R. dominica* activity, whereas the lowest activity, i.e., 10% mortality was observed in *R. dominica* fed with MOL3, MOL6, MOL9, MOL12, and MOL20 (Figure 5). The exudates of remaining isolates were found inactive against the tested insects (Results not shown).

*T. Castaneum* is a global pest found in the stockpiled goods, mainly food grains [48], while *C. analis* beetles specifically grow in the legumes, for example, beans [49], and *R. dominica* is a grain borer of wheat and rice [50]. In general, these pests can severely affect the crop yield and development, so to control such pests, potent pesticides with low effect on the environment are demanded. Shi et al. [51] isolated potent insecticidal compounds from the endophytic fungi *Claviceps purpurea*. Likewise, *Sarocladium strictum* was observed to produce sebacic acid, Penta methoxy flavone, Cis-13-octadecenoic acid, and n-hexadecanoic acid that killed the 2nd larval instar of *S. littoralis* [52].

### 3.8. Larvicidal Activity

**Figure 6:**
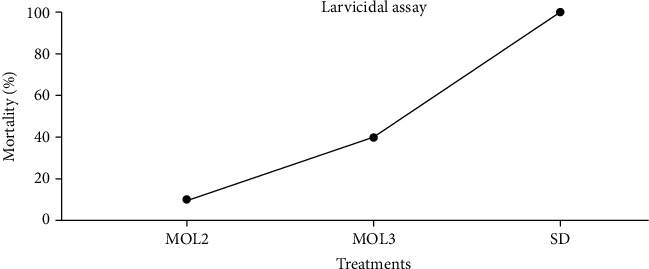
Larvicidal activity to control dengue fever by *M. oleifera* leaves endophytes.

Mosquitoes serve as a vector of many human pathogenic viruses that cause lots of deaths every year around the world [53]. Usage of synthetic insecticides for killing or halting the growth of vector mosquitoes induced physiological resistance [54]. *Ae. Aegypti* is a primary vector of yellow fever and dengue hemorrhagic fever [55]. In the present study, 200 ppm of MOL3 crude extract has killed 40% of *Ae. Aegypti* larvae, while MOL2 extract reduced 10% of the larval population at the same concentration (Figure 6). The extracts of the rest of the isolated endophytes from *M. oleifera* leaves have no impact on the *Ae. Aegypti* larvae (Results not shown). The purified compounds in the extract of MOL3 might play a vital role in controlling yellow fever and dengue in the developed and nondeveloped areas of the world. In recent times, pentane, 1,1,1,5-tetrachloro-preg-4-en-3-one, 17. *α*-hydroxy-17. *β*-cyano-, pentane, 1,1,1,5-tetrachloro-, and trans-3-undecene-1,5-diyne were found in the extract of *Aspergillus tamarii* that exhibited vigorous activity against *Ae. aegypti* and *Culex quinquefasciatus* [56].

### 3.9. Antileishmanial Activity

Three fungal extracts were subjected for leishmanial activity against *Leishmania major* (promastigotes). The results in Table 1 displayed that LC_50_ of MOL3 crude extract was 46.78 *μ*g/ml, while the LC_50_ of MOL1 and MOL2 was greater than 100 *μ*g/ml. The extract of remaining fungal endophytes isolated from *M. oleifera* leaves showed no leishmanial activity (Results not shown). The crude extract of endophytic fungi *Cochliobolus sativus* isolated from the leaves of exhibited antileishmanial activity. Further investigation of the crude extract showed the presence of cochlioquinone A, isocochlioquinone A, and anhydrocochlioquinone A [57]. In Panama, antileishmanial activity was found in the extract of fungus *Edenia* sp., which was attributed to the palmarumycin CP17 and palmarumycin CP18 [58].

## 4. Conclusion

The above study suggested that *M. oleifera* plant is a source of novel yet potent endophytic fungi. The crude extracts of some of the isolated fungal strain from the leaves of *M. oleifera* showed antimicrobial, antifungal, termicidal, phytotoxic, insecticidal, cytotoxic, and anti-inflammatory activities. The compounds in crude extracts of isolates MOL3 and MOL7 can serve as best antibiotics against *E. coli*, while that of MOL6 against *S. aureus*. Similarly, the strains MOL1 and MOL7 can serve as the antifungal agent against *C. albicans* and MOL11 against *V. chlamydosporium*. The isolates, MOL3, MOL7, MOL9, MOL15, MOL17, MOL18, and MOL19, can be used as a source of potent herbicide. MOL2 can be used as a source of cytotoxic, MOL2, MOL6, MOL17, MOL19, and MOL20 as insecticidal, and MOL3 as larvicidal and antileishmanial agents. Moreover, further studies are required on the purified compounds present in the crude exudates of the above mentioned potent strains.

## Figures and Tables

**Table 1:** Antileishmanicidal activity of fungal endophytes isolated from *M. oleifera* leaves against *Leshmania major.*

Test sample	IC50 (*μ*g/ml)
MOL1	>100
MOL2	>100
MOL3	46.78
Amphoterian B	0.39
Pentamidine	3.16

Amphoterian B and Pentamidine use as a positive control.

## Data Availability

All the data are included in the manuscript and supplementary files.

## Supplementary Materials

Supplementary Materials
*Supplementary 1.* Table S1: colony characteristics of endophytic fungi isolated from leaves of *M. oleifera* on PDA. *Supplementary 2.* Table S2: microscopic characteristics of endophytic fungi isolated from leaves of *M. oleifera* on PDA. *Supplementary 3*. Figure S1: micro and macroscopic features of endophytic fungi isolated from the leaves of *M. oleifera*.
