# Supplementary material for: Potent Bioactivity of Endophytic Fungi Isolated from Moringa oleifera Leaves
Source: Biomed Res Int. 2022 Dec 15;2022:2461021. doi: 10.1155/2022/2461021 (PMC9779999; doi:10.1155/2022/2461021)
Supplement: Supplementary Materials — Supplementary 1. Table S1: colony characteristics of endophytic fungi isolated from leaves of M. oleifera on PDA. Supplementary 2. Table S2: microscopic characteristics of endophytic fungi isolated from leaves of M. oleifera on PDA. Supplementary 3. Figure S1: micro and macroscopic features of endophytic fungi isolated from the leaves of M. oleifera. [file 2461021.f1.docx]

**Table S1:** Colony characteristics of endophytic fungi isolated from leaves of *M. oliefera* on PDA

| **Fungal code** | **Name of specie** | **Morphological characteristics of the colony** | | | |
| --- | --- | --- | --- | --- | --- |
|  |  | **Surface** | **Reverse side** | **Growth** | **Margin** |
| **MOL1** | *Penicillium sp.* | Green, velvety | Yellowish to white | Rapid | Irregular |
| **MOL2** | *Fusarium sp* | Pale-white, cottony | Pale | Rapid | Entire |
| **MOL3** | *Aspergillus Niger* | white, quickly becoming black | pale yellow | Fast | Entire |
| **MOL5** | *P. digitatum* | Yellow green turned olive green, velvety | tinted brown | Rapid | Entire |
| **MOL7** | *A. fumigatus* | smoky gray-green, woolly to cottony | Yellow | Rapid | Entire |
| **MOL8** | 1. *flavus* | Olive green, velvety, powdery parrot green spores on surface | Furrowed, pale brown | Rapid | Vary |
| **MOL9** | *Tricoderma sp.* | White, wooly, yellow-green patches after maturation | Pale | Rapid | Filiform |
| **MOL10** | *Rhizoctonia sp.* | White turns to brown | Brown | Fast | Entire |
| **MOL13** | *A. alternata* | Ashy white to light green | Brown | Maximum | Entire |
| **MOL14** | *Mucor sp.* | Yellow, cottony, enter get black with maturation | Wrinkled, orange yellow | Rapid | Irregular |
| **MOL19** | *A. terrus* | Brownish | Deep brown | Rapidly | Entire |
| **MOL21** | *Alternaria cassia* | Pink, cottony | Light reddish | Fast | Wavy |
| **MOL22** | *A. carneus* | Pale yellow-tan | Yellow to red-brown | Fast | Thin |

**Table S2:** Microscopic characteristics of endophytic fungi isolated from leaves of *M. oliefera* on PDA

| **Fungal specie** | **Microscopic characteristics** | | | |
| --- | --- | --- | --- | --- |
|  | **Hyphae** | **Conidiophore** | **Conidia** | **Fruiting body** |
| **MOL1** | Septate | Phialides grouped brush-like clusters | Unicellular, oval, smooth, chains | Cleistothecium |
| **MOL2** | width, septate, branched, haphazardly dispersed | Polyphialidic, loosely branched, short, swollen phialides | Thick macroconidia, hook apical cell | Perithecia |
| **MOL3** | Septate, hyaline | Smooth, hyaline, darker apex | Black, rough, globose | Cliestothecium |
| **MOL5** | Narrow, septate | Asymmetrical, smooth, thin walls, philiades | Cylindrical, chain | Heterothallism |
| **MOL7** | Septate, hyaline | Smooth-walled, uncolored, dome-shaped vesicle | Green echinulate | - |
| **MOL 8** | Septate, thread shape, mycelia, hyaline. | Uncolored, thick walled, coarsely roughened, vesicle bearing | Smooth to fine roughed | Columnar |
| **MOL 9** | Septate | Hyaline, pyramidal arrangement | Green, rough and smooth- walled, grouped in sticky heads | Ellipsoidal |
| **MOL10** | Long tube, septa, partition inside | - | Do not produce | - |
| **MOL13** | septa, straight/ fluxeus | Pale brown to olive brown | Branched, long chain more than 5 conidia. | Ellipsoidal |
| **MOL14** | short,  gametangia | Erect, simple sporangiophore | Multispored sporangia | Ellipsoid to phaseoliform |
| **MOL19** | Septate, hyaline | Smooth, hyaline | Aleurioconidia, globose, smooth-walled, light yellow to hyaline | Compact, biseriate, columna |
| **MOL 21** | Septate | Groups, slightly curved, branched, cylindrical, brown, smooth-walled | Single/2 to 3 chains, Obclavate, murifonn, non-beaked & beaked, secondary conidiophore | - |
| **MOL22** | Irregular hyphal | loosely columnar  with hemispherical vesicle | Smooth, unpigmented spherical | - |

**
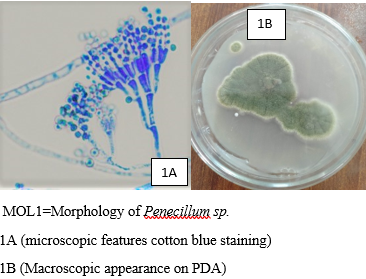

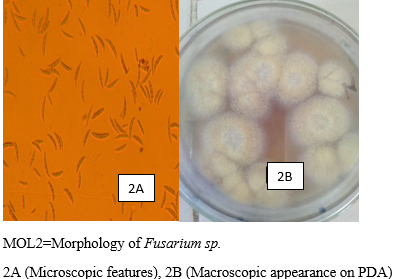
**

**
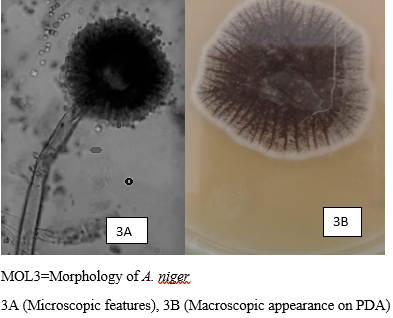

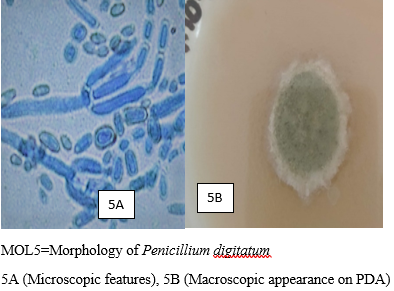
**

**
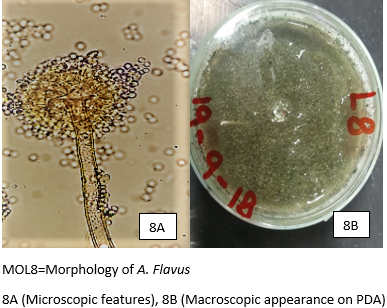

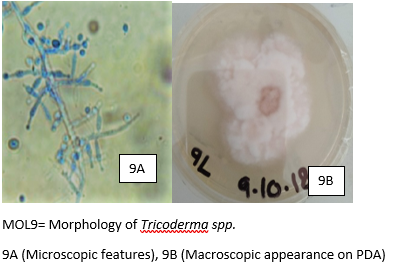
**

**
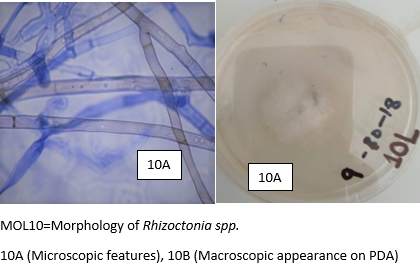

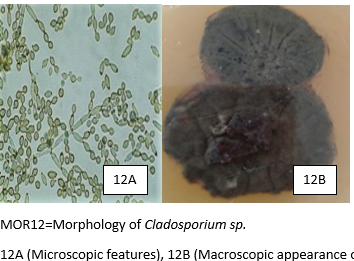
**

**
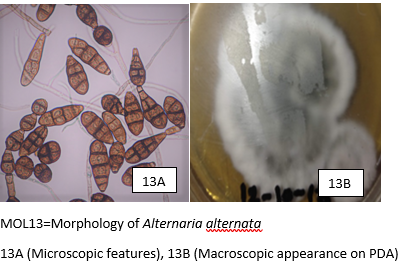

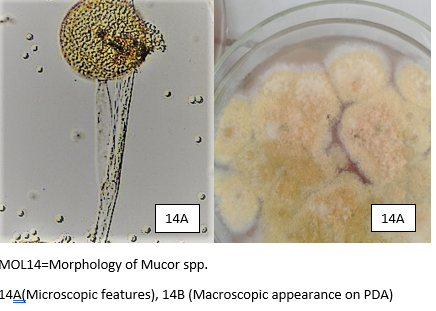
**

**
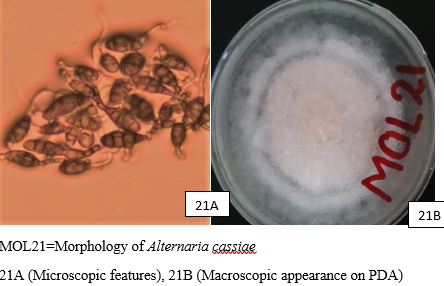

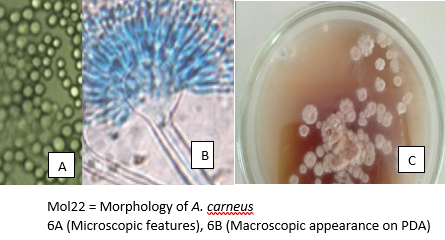
**

**Figure S1:** Micro and Macroscopic features of endophytic fungi isolated from the leaves of *M. oliefera*.
